# Clinicopathological and Molecular Characteristics of *IDH*-Wildtype Glioblastoma with *FGFR3::TACC3* Fusion

**DOI:** 10.3390/biomedicines12010150

**Published:** 2024-01-10

**Authors:** Hyunsik Bae, Boram Lee, Soohyun Hwang, Jiyeon Lee, Hyun-Soo Kim, Yeon-Lim Suh

**Affiliations:** 1 Pathology Center, Seegene Medical Foundation, Seoul 04805, Republic of Korea; hbae0827@gmail.com; 2 Department of Pathology and Translational Genomics, Samsung Medical Center, Sungkyunkwan University School of Medicine, Seoul 06351, Republic of Korea; boram.lee@samsung.com (B.L.); juzzsaw@naver.com (S.H.); 3 Department of Pathology, Guro Hospital, Korea University College of Medicine, Seoul 08308, Republic of Korea; jiyeon_l@naver.com

**Keywords:** adult-type diffuse glioma, glioblastoma, *IDH*-wildtype, *FGFR3::TACC3* fusion

## Abstract

The World Health Organization Classification of Tumors of the Central Nervous System recently incorporated histological features, immunophenotypes, and molecular characteristics to improve the accuracy of glioblastoma (GBM) diagnosis. *FGFR3::TACC3* (F3T3) fusion has been identified as an oncogenic driver in *IDH*-wildtype GBMs. Recent studies have demonstrated the potential of using FGFR inhibitors in clinical trials and TACC3-targeting agents in preclinical models for GBM treatment. However, there is limited information on the clinicopathological and genetic features of *IDH*-wildtype GBMs with F3T3 fusion. The aim of this study was to comprehensively investigate the clinical manifestations, histological features, and mutational profiles of F3T3-positive GBMs. Between September 2017 and February 2023, 25 consecutive cases (5.0%) of F3T3-positive GBM were extracted from 504 cases of *IDH*-wildtype GBM. Clinicopathological information and targeted sequencing results obtained from 25 primary and 4 recurrent F3T3-positive GBMs were evaluated and compared with those from F3T3-negative GBMs. The provisional grades determined by histology only were distributed as follows: 4 (26/29; 89.7%), 3 (2/29; 6.9%), and 2 (1/29; 3.4%). Grade 2–3 tumors were ultimately diagnosed as grade 4 GBMs based on the identification of the *TERT* promoter mutation and the combined gain of chromosome 7 and loss of chromosome 10 (7+/10−). F3T3-positive GBMs predominantly affected women (2.6 females per male). The mean age of patients with an F3T3-positive GBM at initial diagnosis was 62 years. F3T3-positive GBMs occurred more frequently in the cortical locations compared to F3T3-negative GBMs. Imaging studies revealed that more than one-third (12/29; 41.4%) of F3T3-positive GBMs displayed a circumscribed tumor border. Seven of the seventeen patients (41.2%) whose follow-up periods exceeded 20 months died of the disease. Histologically, F3T3-positive GBMs more frequently showed curvilinear capillary proliferation, palisading nuclei, and calcification compared to F3T3-negative GBMs. Molecularly, the most common alterations observed in F3T3-positive GBMs were *TERT* promoter mutations and 7+/10−, whereas amplifications of *EGFR*, *PDGFRA*, and *KIT* were not detected at all. Other genetic alterations included *CDKN2A/B* deletion, *PTEN* mutation, *TP53* mutation, *CDK4* amplification, and *MDM2* amplification. Our observations suggest that F3T3-positive GBM is a distinct molecular subgroup of the *IDH*-wildtype GBM. Both clinicians and pathologists should consider this rare entity in the differential diagnosis of diffuse astrocytic glioma to make an accurate diagnosis and to ensure appropriate therapeutic management.

## 1. Introduction

The 2016 World Health Organization (WHO) Classification of Tumors of the Central Nervous System (CNS) incorporated histological features and immunophenotypes to improve the accuracy of glioblastoma (GBM) diagnosis [1]. With the integration of the next-generation sequencing (NGS) technique into the diagnostic process, novel molecular characteristics of GBMs have been discovered. In the 2021 WHO Classification of Tumors of the CNS, the adoption of the Consortium to Inform Molecular and Practical Approaches to CNS Tumor Taxonomy (cIMPACT-NOW) further improved the classification of gliomas, especially aiding in prognosis prediction [2].

Isocitrate dehydrogenase (*IDH*)-wildtype glioblastoma (GBM) is a highly heterogeneous high-grade glioma [3]. The molecular diagnosis of *IDH*-wildtype GBM can be established based on the following three molecular parameters: the combined gain of chromosome 7 and loss of chromosome 10 (7+/10−), epidermal growth factor receptor (*EGFR*) amplification, and telomerase reverse transcriptase (*TERT*) promoter mutation [2]. Even though research on oncogenic driver mutations applicable to specific targeted therapies for GBMs is actively ongoing, their rarity has limited their use in treatment.

Gene fusion refers to the hybridization of coding or regulatory DNA sequences between genes resulting from genomic rearrangements such as translocations, deletions, duplications, and inversions [4]. The fusion between fibroblast growth factor receptor 3 (*FGFR3*) and transforming acidic coiled-coil-containing protein 3 (*TACC3*; F3T3 fusion) has been recently identified as an oncogenic driver in 3% of *IDH*-wildtype GBMs [5]. This fusion gene encodes a protein located at the poles of the mitotic spindle and exhibits hyperphosphorylation and constitutive activation of the kinase domain [6]. *IDH*-wildtype GBMs with F3T3 fusion (F3T3-positive GBMs) have been shown to exhibit distinct histological and molecular features [6,7,8], including the palisading of monotonous, ovoid nuclei; the curvilinear proliferation of thin capillaries; calcification; desmoplastic vessels; and amplifications of cyclin-dependent kinase 4 (*CDK4*) and mouse double minute 2 homolog (*MDM2*) [9,10,11]. It has been also reported that patients with an F3T3-positive GBM show better prognoses than those with an F3T3-negative GBM [5,9,11]. Some studies have highlighted differences in methylation profiles, tumor mutational burdens (TMBs), and copy number alterations between F3T3-positive and -negative GBMs [5,12].

Some previous studies have been conducted on F3T3-positive GBMs from multi-institutional cohorts [8,13], but their clinicopathological features remain to be clarified. In this study, we aimed to comprehensively analyze the clinical manifestations, imaging findings, histological features, and molecular characteristics of 25 consecutive cases of F3T3-positive GBM diagnosed at a single institution.

## 2. Materials and Methods

### 2.1. Patient Selection

Between September 2017 and February 2023, 25 cases of diffuse astrocytic gliomas with F3T3 fusion were extracted from a cohort of 504 *IDH*-wildtype GBM patients (5.0%) using next-generation sequencing (NGS) analysis. Twenty-five and four tumors were diagnosed as primary and recurrent F3T3-positive GBMs, respectively. Among the 479 patients with *IDH*-wildtype GBM without F3T3 fusion, 40 were randomly selected to establish an age-matched control group using the simultaneously conducted NGS analysis. Clinicopathological and demographic information was collected from the electronic medical records.

### 2.2. Histological and Neuroimaging Analysis

Two board-certified neuropathologists (H.B. and Y.-L.S.) independently reviewed all available hematoxylin and eosin-stained slides to ensure consistency and accuracy in the assessment of histological features. The following histological features were assessed: curvilinear capillary proliferation, nuclear palisading, calcification, palisading necrosis, desmoplastic vessels, tumor giant cells, and perivascular rosette formation. They also reviewed all available brain magnetic resonance images (MRIs) and the corresponding radiological interpretations.

### 2.3. NGS

Formalin-fixed, paraffin-embedded tissues were microdissected manually on unstained sections under a microscope. Genomic DNA was extracted using the QIAamp DNA FFPE Tissue Kit (Qiagen, Hilden, Germany). The DNA samples were fragmented using the Covaris S2 instrument (Covaris, Woburn, MA, USA), and a library was prepared using the SureSelect XT Automation Reagent Kit (Agilent Technologies, Santa Clara, CA, USA). To ensure the appropriate fragment size, the median size of the DNA fragments was determined using the Agilent 4200 Tape Station (Agilent, Santa Clara, CA, USA), with the desired range set between 300 and 400 bp. The concentration of the DNA was measured using the Qubit dsDNA High Sensitivity Assay Kit (Thermo Fisher Scientific, Waltham, MA, USA). Sequencing was performed on the NextSeq 500/550 platform using the TG NextSeq 500/550 High Output Kit v2.0 and the TG NextSeq 500/550 Mid Output Kit v2.0 (NextSeq 550 Dx, Illumina, San Diego, CA, USA). Data analysis and interpretation were conducted using BrainTumorSCAN v2.0 (Geninus, Seoul, Republic of Korea). The BrainTumorSCAN (Geninus) pipeline consisted of a sequencing panel targeting 233 glioma-related genes. The obtained sequence data were analyzed to identify clinically relevant genomic alterations, including single-nucleotide variants (SNVs), small insertions/deletions (indels), copy number alterations (CNAs), and rearrangements/fusions. SNVs and indels with a variant allele frequency (VAF) below 1% were excluded from the analysis. Copy number gains were defined as having an average copy number greater than four, while losses were defined as having a copy number less than one. Translocations were identified based on supporting reads ranging from four to twelve, depending on the sample quality. The NGS results were manually reviewed by four board-certified pathologists (H.B., B.L., S.H., and Y.-L.S.) and one bioinformatician (D.G.K.). Reported variants were cross-referenced with ClinVar (https://www.ncbi.nlm.nih.gov/clinvar/, accessed on 1 December 2023) to exclude variants that were reported as benign or likely benign. The genomic data analysis visualization was performed using the cBioPortal Oncoprinter (https://www.cbioportal.org/oncoprinter, accessed on 1 December 2023).

### 2.4. Statistical Analysis

Statistical analysis was performed using IBM SPSS Statistics for Windows, v27.0 (IBM Corporation, Armonk, NY, USA). Categorical variables, including clinical features, histological features, and imaging findings, were compared between F3T3-positive and -negative GBMs using Fisher’s exact test or the chi-square test. Overall survival was defined as the time from initial diagnosis to death, with there being censored observations for patients who were still alive at the last follow-up. Survival curves were generated using the Kaplan–Meier method, and differences between the curves were assessed using the log-rank test. Statistical significance was defined as a *p* < 0.05.

## 3. Results

### 3.1. Demographics

**Figure 1:**
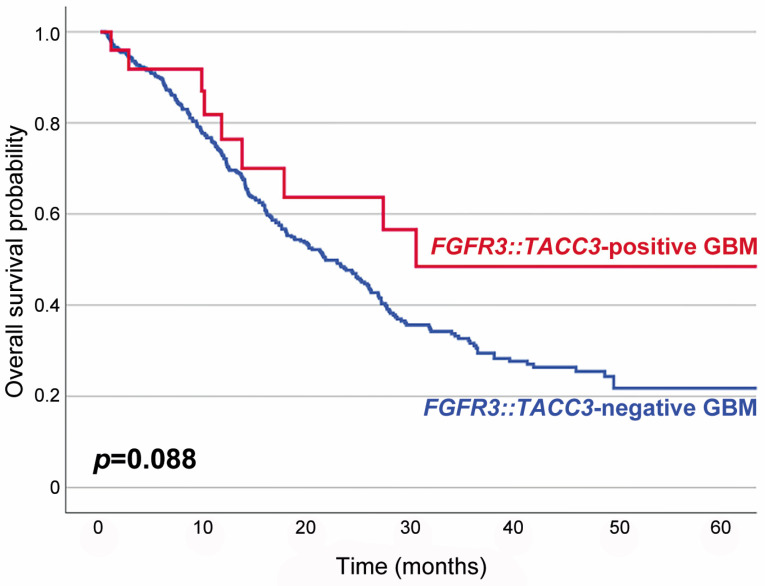
Kaplan–Meier plots showing the overall survival probabilities of patients with F3T3-positive GBM (blue line) and those with F3T3-negative GBM (red line). The overall survival rate of F3T3-positive GBM patients was higher than that of F3T3-negative GBM patients, but the difference was not statistically significant (*p* = 0.088).

Table 1 summarizes the association between F3T3 fusion and the clinicopathological characteristics of *IDH*-wildtype GBMs. Twenty-five primary and four recurrent tumor specimens were obtained from 25 patients. F3T3-positive GBMs predominantly affected women (2.6 females per male; *p* = 0.003). The mean age of patients with an F3T3-positive GBM at initial diagnosis was 62 years (median age, 60 years; range, 40–84 years), which was similar to that of the 479 patients with an F3T3-negative GBM (60 years; *p* = 0.424; range, 19–87 years). Seventeen of the twenty-nine tumors (58.6%) presented as a single lesion and were totally excised. The remaining 12 tumors (41.4%) showed multiple lesions and were diagnosed with navigation-guided stereotactic biopsies. Nineteen patients (76.0%) received concurrent chemoradiation therapy with temozolomide, while two (8.0%) received radiation therapy only. The mean follow-up period for the 25 patients with an F3T3-positive GBM was 19 months (median, 12 months; range, 3–69 months). Fifteen patients (60.0%) developed tumor recurrence or progression during the follow-up period. Six of the seventeen patients whose follow-up period exceeded 20 months died of disease. Eight patients with a follow-up period shorter than 20 months were alive at the last follow-up. Survival analysis revealed that patients with an F3T3-positive GBM had a longer overall survival than those with an F3T3-negative GBM, but the difference in survival rate between the two groups was not statistically significant (*p* = 0.088; Figure 1).

### 3.2. Histological Analysis

**Figure 2:**
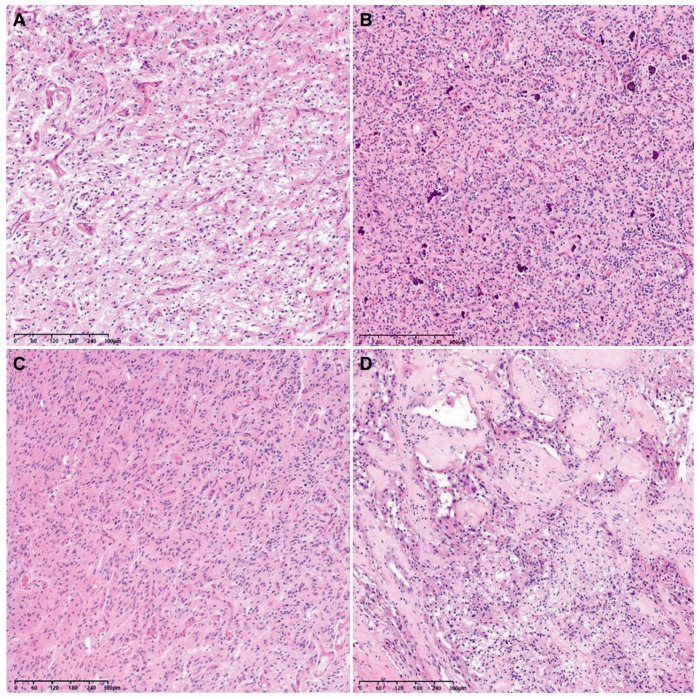
Representative photomicrographs showing the histological features of an F3T3-positive GBM. The tumor tissue exhibits curvilinear capillary proliferation (**A**), scattered calcification within the tumor (**B**), nuclear palisading (**C**), and desmoplastic vessels (**D**).

The histological grades of 29 tumor tissues were distributed as follows: 26 were grade 4 (89.7%), 2 were grade 3 (6.9%), and 1 was grade 2 (3.4%). Since the lower-grade tumors displayed neither tumor cell necrosis nor microvascular proliferation, their provisional grade was grade 2 or 3. However, they were finally confirmed as WHO grade 4 GBMs based on their NGS results showing either a *TERT* promoter mutation (1/3), 7+/10− (1/3), or both (1/3; Table 2). Figure 2 depicts representative photomicrographs showing the histological features of an F3T3-positive GBM.

As shown in Table 3, the most common histological feature observed in the F3T3-positive group was curvilinear capillary proliferation (25/29; 86.2%), followed by desmoplastic vessels (18/29; 62.1%), palisading nuclei (16/29; 55.2%), palisading necrosis (14/29; 48.3%), and calcification within the tumor (13/29; 44.8%). Tumor giant cells and a perivascular rosette pattern were observed in 10 (34.5%) and 8 (27.6%) cases of F3T3-positive GBM, respectively. Compared to the F3T3-negative GBMs, F3T3-positive tumors showed curvilinear capillary proliferation (*p* < 0.001), nuclear palisading (*p* = 0.001), and calcification (*p* = 0.006) more frequently. The frequencies of desmoplastic vessels (*p* = 0.429), palisading necrosis (*p* = 0.429), tumor giant cells (*p* = 0.271), and perivascular rosette formation (*p* = 0.114) were not significantly different between F3T3-positive and -negative GBMs.

### 3.3. Neuroimaging Analysis

As shown in Table 4, F3T3-positive GBMs were most frequently found in the right cerebral hemisphere (11/29; 37.9%) and the frontal lobe (16/29; 55.2%). These tumors were not found in the deep nuclei or cerebellum. Ten tumors (34.5%) were found in the bilateral cerebral hemispheres. In 12 cases (41.4%), the tumors had multiple anatomical locations. In contrast, F3T3-negative GBMs occurred most commonly in the left cerebral hemisphere (19/40; 47.5%) and the frontal lobe (20/40; 50.0%). While the tumor laterality and location were not significantly different according to the status of F3T3 fusion, cortical localization of tumors was significantly more frequent in F3T3-positive GBMs than in tumors without F3T3 (*p* = 0.006). Twelve cases of F3T3-positive GBM (41.4%) showed relatively well-circumscribed tumor borders, but there was no statistical significance in tumor border configuration between the two groups (*p* = 0.093).

### 3.4. Molecular Characteristics

**Figure 3:**
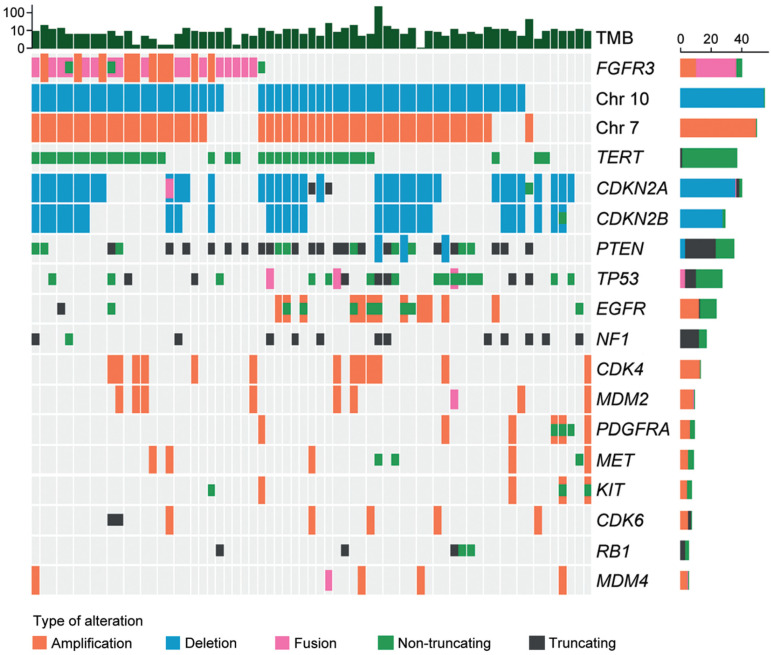
Molecular characteristics of F3T3-positive GBMs.

Twenty-seven tumor tissue samples were available for NGS. As shown in Figure 3, the most common fusion type was identified between *FGFR3* exon 17 and *TACC3* exon 11 (7/27; 25.9%), followed by the fusion between *FGFR3* exon 18 and *TACC3* exon 11 (6/27; 22.2%). The most common exon was exon 18 in *FGFR3* (15/27; 55.6%) and exon 11 in *TACC3* (13/27; 48.1%). All fusions were in-frame fusions, encoding a structural protein with consistent tyrosine kinase activity. The mean TMB of F3T3-positive GBMs was 7.08 per megabase (range of 1.51–19.67 per megabase). The most common genetic alterations detected in F3T3-positive GBMs were 7+/10− (22/27; 81.5%), followed by the *TERT* promoter mutation (19/27; 70.4%). None of the F3T3-positive GBMs demonstrated amplifications in *EGFR*, platelet-derived growth factor receptor A (*PDGFRA*), or *KIT*. Other alterations included CDK inhibitor 2A (*CDKN2A*) deletion (13/27; 48.1%), CDK inhibitor 2B (*CDKN2B*) deletion (10/27; 37.0%), phosphatase and tensin homolog deletion in chromosome 10 (*PTEN*) mutation (9/27; 33.3%), *CDK4* amplification (6/27; 22.2%), *TP53* mutation (5/27; 18.5%), and murine double minute 2 (*MDM2*) amplification (4/27; 14.8%). The information on O-6-methylguanine-DNA methyltransferase (*MGMT*) promoter methylation was available in 25 F3T3-positive GBMs. Thirteen F3T3-positive tumors (52.0%) had a methylated *MGMT* promoter.

Table 5 summarizes the difference in the frequencies of genetic alterations between F3T3-positive and -negative GBMs. In total, 28 and 17 of the 40 F3T3-negative GBMs had 7+/10− and the *TERT* promoter mutation, respectively. There was no significant difference in the alteration frequencies according to the presence of F3T3 fusion (*p* = 0.842 and *p* = 0.773). The *EGFR* and *PDGFRA* amplifications were detected in 12 (*p* = 0.002) and 6 (*p* = 0.035) F3T3-negative GBMs, respectively. There were no significant differences in the frequencies of other mutations and the degree of *MGMT* promoter methylation between the two groups.

### 3.5. Brief Presentation of a Case of F3T3-Positive GBM Showing Unusual Histology

**Figure 4:**
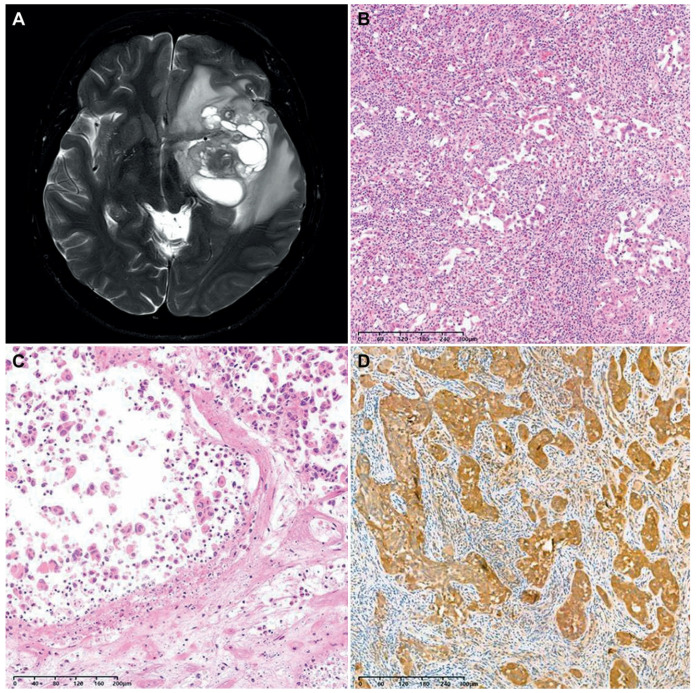
A case of F3T3-positive GBM showing unusual histology. (**A**) The brain MRI revealed a relatively well-circumscribed, multiloculated cystic mass in the left temporal lobe. (**B**) The tumor cells were arranged in an alveolar or micropapillary pattern. The peritumoral area exhibited mixed inflammatory infiltrates, including eosinophils. (**C**) In some microscopic foci, individually dispersed tumor cells displayed a cell-in-cell phenomenon. (**D**) Immunostaining revealed that the tumor cells diffusely and strongly expressed the S100 protein.

We encountered a case of an F3T3-positive GBM displaying unusual histology. A 40-year-old woman presented with a 6.7 cm sized multiloculated mass in the left temporal lobe (Figure 4A). She underwent gross total resection. The tumor exhibited a relatively well-circumscribed border. Histologically, the tumor consisted of round-to-oval cells arranged in an alveolar or micropapillary pattern (Figure 4B). The tumor cells possessed pleomorphism nuclei with vesicular chromatin, conspicuous nucleoli, and an eosinophilic cytoplasm. The peritumoral area showed significant inflammatory infiltrates with eosinophils. The tumor lacked the characteristic histological features typically seen in F3T3-positive GBMs. Instead, tumor cell necrosis, frequent mitotic figures, and a cell-in-cell phenomenon were observed (Figure 4C). Immunostaining revealed that the tumor cells were negative for glial fibrillary acidic protein and oligodendrocyte transcription factor 2, but they were diffusely positive for CD56 and S100 protein (Figure 4D). NGS analysis revealed the presence of 7+/10−, F3T3 fusion, and amplifications of *CDK4* and *MDM2*, compatible with the diagnosis of *IDH*-wildtype GBM with F3T3 fusion.

## 4. Discussion

Diffuse gliomas are categorized into three groups: adult-type diffuse gliomas, pediatric-type diffuse low-grade gliomas, and pediatric-type diffuse high-grade gliomas [8]. Extensive molecular research has been conducted in the field of brain tumors to achieve an integrated diagnosis, leading to a comprehensive understanding of the molecular landscape [14,15,16]. Even though there is a pressing need for novel drug research specific to brain tumors, patients with a GBM still present dismal prognoses and often develop resistance to conventional therapy [5]. Clinical trials targeting several oncogenic mutations and cellular signaling pathways frequently observed in GBMs, such as *EGFR* mutation or amplification, the phosphoinositide 3-kinase/Akt/mammalian target of the rapamycin pathway, the p53 pathway, and the retinoblastoma (RB) pathway, have failed to improve outcomes due to challenges related to blood–brain barrier penetration, drug stability, and safety concerns [17]. While *FGFR* alterations are frequently observed in GBMs, their clinical significance may be limited since therapeutically meaningful alterations, such as F3T3 fusion, are rare [18].

FGFR proteins modulate various signaling pathways through molecular alterations and have significant importance in cancer biology. Multiple FGFR inhibitors currently in clinical development demonstrate therapeutic relevance and scalability [19]. The *TACC3* gene has been identified as an important partner for *FGFR* fusions and is associated with the pathogenesis of several solid tumors [19]. The TACC3 protein possesses a coiled-coil domain at its C terminus, which promotes stability and organization of the mitotic spindle [20]. Since F3T3 fusion was first reported in GBMs and urothelial carcinomas of the urinary bladder [21,22], it has now been observed in pulmonary adenocarcinoma and squamous cell carcinoma, nasopharyngeal carcinoma, and uterine cervical squamous cell carcinoma [23,24,25,26,27]. The mechanism of action of this fusion protein involves delaying mitotic progression and inducing aneuploidy by increasing downstream signaling through the mitogen-activated protein kinase pathway, activating mitochondrial biogenesis and metabolism, and recruiting endogenous TACC3 from the mitotic spindle. These processes are believed to enhance malignant transformation [19,28,29]. The discovery of oncogenic gene fusions has significant clinical implications, suggesting the possibility of targeted therapy. A personalized approach utilizing *FGFR* fusion-targeted inhibitors may improve the prognosis of patients with gliomas [30]. Tabernero et al. [31] reported partial responses among patients with F3T3-positive tumors who were treated with the *FGFR* inhibitor erdafitinib. Several targeted therapies have been evaluated in clinical trials for patients with gliomas with *FGFR* fusion, including erdafitinib, ponatinib, and infigratinib [1]. These studies indicated that F3T3 fusion is a potential therapeutic target. Particularly, tumors with F3T3 fusion appear to be more sensitive to *FGFR*-targeted therapy compared to those with other *FGFR* abnormalities. This finding is meaningful because treatment options for patients with aggressive tumors with F3T3 fusion, such as a GBM and urothelial carcinoma, are limited [32].

F3T3 fusion has been detected in 3–12% of adult GBM cases [5,11,12,33]. Consistent with previous data, in this study, we detected F3T3 fusion in 5.0% (25/504) of the *IDH*-wildtype GBMs. Notably, we observed a higher frequency of F3T3 fusion in women (72.0%) compared to men (28.0%). When compared to F3T3-negative GBMs, a significant female predilection was observed (2.6 females per male; *p* = 0.003). In contrast, three previous studies reported either equal rates of F3T3 fusion in men and women [9] or slightly higher rates in men (1.47 male per female and 1.2 male per female) [5,8]. Since the previous studies showing equal gender distribution were conducted in Western populations, we considered that the difference in gender distribution may reflect racial difference. It is reasonable to assume that the relative incidence of F3T3-positive GBM may vary by ethnicity. Both domestic multi-institutional and global multinational studies are necessary to confirm or disprove this assumption and to explore the underlying factors contributing to this gender-based difference in the occurrence of F3T3 fusion.

Our study provides the first evidence that GBMs with F3T3 fusion are more frequently located in the cerebral cortical area compared to F3T3-negative tumors. Twelve of the twenty-nine F3T3-positive GBMs (41.3%) were located in the cerebral cortex or subcortical area. Consistent with our results, Di Stefano et al. [30] investigated the radiological profiles of gliomas with F3T3 fusion and found they more often affected the insula and temporal lobe. In contrast, Roux et al. [34] analyzed MRI scans of 392 GBM patients with the *IDH* wildtype and reported 63.3% (248/392) of GBMs were located in the subcortical white matter of the subventricular zones of both hemispheres. Since they did not mention the presence of absence of F3T3 fusion in their cases, we cannot determine whether they examined F3T3-positive tumors, F3T3-negative tumors, or a mixture of both types.

Bielle et al. [9] reported that F3T3-positive GBMs are histologically characterized by monomorphic, ovoid nuclei; nuclear palisading; a curvilinear capillary network; calcification; and desmoplastic vessels. We found that curvilinear capillary networks (*p* < 0.001), nuclear palisading (*p* = 0.001), and calcification (*p* = 0.004) were more frequently identified in F3T3-positive GBMs compared to the control group. In cases where these three recurrent characteristic histological findings are encountered in the diagnosis of *IDH*-wildtype GBM, pathologists should consider the possibility of an F3T3 fusion and perform a molecular test. Additionally, FGFR3 immunostaining has been recommended as a screening tool for detecting F3T3 fusion, with a sensitivity of 100% and a specificity of 92% [9]. In F3T3-positive cases, FGFR3 immunostaining consistently revealed a positive expression, although heterogeneous staining patterns were occasionally observed. Particularly, areas exhibiting the characteristic histological features exhibited a stronger staining intensity [9]. Therefore, FGFR3 immunostaining can facilitate the efficient prediction of F3T3 fusion, making the diagnostic process easier.

We found that none of the *IDH*-wildtype GBMs with F3T3 fusion had *EGFR* amplification, which is consistent with the findings of a previous study by Di Stefano et al. [11]. These observations suggest that F3T3 fusion is mutually exclusive with *IDH* mutation and *EGFR* amplification. Similarly, none of the F3T3-positive GBMs presented with *KIT* or *PDGFRA* amplifications, while 83 (17.4%) and 63 (13.2%) of the 479 F3T3-negative GBMs had *PDGFRA* and *KIT* amplifications, respectively. The absence of the amplification of these genes in our F3T3-positive GBM cases raises the possibility of mutual exclusivity between *KIT/PDGFRA* and F3T3 fusion, similar to that of *IDH* mutation and *EGFR* amplification. Consistent with our data, Mata et al. [5] also reported a lack of *PDGFRA* and *KIT* amplifications in their F3T3-positive GBMs, demonstrating significant differences from that of F3T3-negative GBMs. In contrast, according to the 2021 WHO Classification of Tumors of the CNS, *PDGFRA* alteration has been reported in 10–15% of GBM cases [14,21,35]. Nobusawa et al. [36] observed the amplifications of *PDGFRA*, *KIT*, and *KDR* in 33 (8.5%), 17 (4.4%), and 13 (3.3%) of 390 GBMs, respectively. Further studies are warranted to investigate whether the absence of *EGFR*, *PDGFRA*, and *KIT* amplifications is due to the rarity of reported GBM cases with F3T3 fusion or is due to these alterations and F3T3 fusion being mutually exclusive events. We found no significant difference in the frequency of TP53 alteration between the F3T3-positive and -negative groups. Even though we further analyzed whether the difference exists according to the type of *TP53* mutation, Fisher’s exact test revealed that non-truncating mutation (*p* = 0.102), truncating mutation (*p* = 0.583), and fusion (*p* = 0.372) of *TP53* did not show any statistical significance. Further investigations using a larger cohort are necessary to compare both the mutational profiles of *TP53* and the expression patterns of p53 protein between F3T3-positive and -negative GBMs.

Unlike the previous reports by Bielle et al. [9] and Di Stefano et al. [11], this study represents a large series of 25 consecutive F3T3-positive GBM cases diagnosed in a single institution. We comprehensively analyzed a wide range of clinicopathological characteristics, including clinical features, radiological characteristics, histological findings, and molecular characteristics. Bielle et al. [9] described a multi-institutional series of 30 patients with diffuse gliomas, including low-grade (grade 2) tumors. They demonstrated recurrent morphological features and molecular characteristics. Similarly, Di Stefano et al. [11] collected 80 F3T3-positive glioma cases from multiple institutions encompassing low-grade (grades 2 and 3) gliomas. They investigated the radiological features and molecular characteristics. In this study, by combining the clinical, pathological, and imaging features, we provided strong, unique insights into understanding the implications of F3T3 fusion in *IDH*-wildtype GBMs.

In summary, our findings suggest that F3T3-positive GBM is a distinct molecular subgroup within the *IDH*-wildtype GBM group. Both clinicians and pathologists should consider this rare entity in the differential diagnosis of diffuse astrocytic glioma to make an accurate diagnosis and to ensure appropriate therapeutic management. Identifying this rare entity based on its distinctive imaging findings and histological features allows for the potential utilization of FGFR3 inhibitors or TACC3-targeting agents, opening up additional treatment options for patients. As clinical trials investigating novel therapeutic agents progress, it will be meaningful to assess their efficacy in F3T3-positive GBMs and to compare their impact on survival rates with those in the non-fusion group.

## Figures and Tables

**Table 1:** Association between F3T3 fusion and the clinicopathological characteristics of *IDH*-wildtype GBMs.

Characteristic		Number of Cases (%)		*p* Value
		F3T3-Positive GBM	F3T3-Negative GBM	
Sex	Male	7 (28.0)	282 (58.9)	0.003 *
	Female	18 (72.0)	197 (41.1)	
Mean age (range; years)		62 (40–84)	60 (19–87)	0.424
Tumor laterality	Single	17 (58.6)	374 (78.1)	0.006 *
	Multiple	12 (41.4)	105 (21.9)	
Type of procedure	Resection	17 (58.6)	380 (79.3)	0.004 *
	Biopsy	12 (41.4)	99 (20.7)	
Overall survival	Mean (months)	23	19	0.088
	Alive	19 (76.0)	214 (44.7)	
	Dead	6 (24.0)	265 (55.3)	

* Statistically significant.

**Table 2:** Histological and molecular grades of F3T3-positive GBMs.

Histological Grade	Number of Cases (%)	Molecular Feature			
		*TERT* Promoter Mutation Only	7+/10− Only	Both *TERT* Promoter Mutation and 7+/10−	Absent
2	1 (3.4)	1	0	0	0
3	2 (6.9)	0	1	1	0
4	26 (89.7)	3	6	16	1

**Table 3:** Association between F3T3 and the histological features of IDH-wildtype GBMs.

Characteristic	Number of Cases (%)		*p* Value
	F3T3-Positive GBM (*n* = 29)	F3T3-Negative GBM (*n* = 40)	
Curvilinear capillary proliferation	25 (86.2)	6 (15.0)	<0.001 *
Desmoplastic vessels	18 (62.1)	21 (52.5)	0.429
Nuclear palisading	16 (55.2)	6 (15.0)	0.001 *
Palisading necrosis	14 (48.3)	16 (40.0)	0.494
Calcification	13 (44.8)	6 (15.0)	0.006 *
Tumor giant cells	10 (34.5)	9 (22.5)	0.271
Perivascular rosettes	8 (27.6)	5 (12.5)	0.114

* Statistically significant.

**Table 4:** Association between F3T3 fusion and the locational information of *IDH*-wildtype GBMs.

Characteristic		Number of Cases (%)		*p* Value
		F3T3-Positive GBM (*n* = 29)	F3T3-Negative GBM (*n* = 40)	
Laterality	Right	11 (37.9)	15 (37.5)	0.971
	Left	8 (27.6)	19 (47.5)	0.094
	Bilateral	10 (34.5)	6 (15.0)	0.058
Location	Frontal lobe	16 (55.2)	20 (50.0)	0.671
	Parietal lobe	9 (31.0)	7 (17.5)	0.189
	Temporal lobe	8 (27.6)	15 (37.5)	0.389
	Occipital lobe	2 (6.9)	2 (5.0)	0.739
Epicenter	Cortex	5 (17.2)	0 (0.0)	0.006 *
	Subcortex	7 (24.1)	3 (7.5)	0.053
	White matter	17 (58.7)	37 (92.5)	0.001 *
Border	Circumscribed	12 (41.4)	9 (22.5)	0.093
	Infiltrative	17 (58.6)	31 (77.5)	0.179

* Statistically significant.

**Table 5:** Differences in the frequencies of genetic alterations between F3T3-positive and -negative GBMs.

Alteration	Number of Cases (%)		*p* Value
	F3T3-Positive GBM (*n* = 27)	F3T3-Negative GBM (*n* = 40)	
7+/10−	22 (81.5)	28 (70.0)	0.842
*TERT* promoter mutation	19 (70.4)	17 (42.5)	0.773
*EGFR* amplification	0 (0.0)	12 (30.0)	0.002 *
*PDGFRA* amplification	0 (0.0)	6 (15.0)	0.035 *
*KIT* amplification	0 (0.0)	4 (10.0)	0.142
*CDKN2A* deletion	13 (48.1)	23 (57.5)	0.211
*CDKN2B* deletion	10 (37.0)	18 (45.0)	0.211
*PTEN* mutation	9 (33.3)	24 (60.0)	0.046 *
*CDK4* amplification	6 (22.2)	7 (17.5)	0.755
*TP53* mutation	5 (18.5)	17 (42.5)	0.105
*MDM2* amplification	4 (14.8)	4 (10.0)	0.705
*MGMT* promoter methylation	13 (52.0)	12 (30.0)	0.310

* Statistically significant.

## Data Availability

No new data were created or analyzed in this study. Data sharing is not applicable to this article.
